# Progress in Rhinology

**Published:** 1895-10-26

**Authors:** 


### PROGRESS IN RHINOLOGY.

**Turbinal Hypertrophy.**—The literature of this subject has received some interesting contributions recently. A succinct account of the microscopic appearances of mulberry growths of the inferior turbinate was given by Wyatt Wingrave in his paper at the Bristol meeting of the British Medical Association, 1894, when he proposed the designation "turbinal varix." The description of the histology of these common sources of nasal obstruction, as given by this author, was as follows[1]: "The peculiar villous or brain-like appearance of the surface was seen microscopically to correspond with a cystic invagination of the surface epithelium, covering distended loops of vessels with very thin walls embedded in mucoid tissue—that is, connective tissue in which the matrix was in excess of the fibrous reticulum and cells. The muscular walls presented well-marked atrophy and degeneration, varying from simple thinning to complete disappearance, owing to the fibres apparently sharing the surrounding mucoid and œdematous changes. In places the intervening mucoid tissue simply formed their boundaries, whilst in other parts the walls seemed to have undergone fibrotic changes. This condition is, therefore, not a mere hypertrophy of the structures, but consists of a true degeneration and infiltration of the walls of these vascular spaces, a morbid process which is responsible for the disease; for the walls gradually losing their power of active recoil, the vessels by degrees become more and more distended, and a permanent enlargement ensues, which is, in fact, a varix."

**Moriform Hypertrophy of the Inferior Turbinated Bodies.**—Jonathan Wright, of Brooklyn,[2] has described and figured these moriform hypertrophies in a paper which was intended to once more draw attention to the distinction existing between these bodies and true papillomata, a point which is constantly being overlooked by specialists, many of whom, on examining a case of nasal obstruction, and observing these foliated masses projecting into the inferior meati, seem incapable of persuading themselves that they have not true papillomata presented before them. These latter, though rare, are occasionally observed growing from the floor and lower portion of the septum nasi, but it is doubtful if one has ever been seen growing from the inferior turbinate body itself. Wright endeavours to account for the papilloma-like external appearance of moriform hypertrophies in these words: "The cause of this folding and crumpling, I conjecture, is the continued and exaggerated contraction and dilatation of the venous sinuses in a stroma deprived by chronic inflammation of much of its elastic and muscular fibres, and having in their place a considerably larger amount of non-contractile fibrous tissue. This exaggerated vaso motor action is indicated in the clinical history these patients give of varying degrees of nasal stenosis." Wright also points out that almost every fold communicates (as seen in his illustration) with the central mass, and that there is no appearance of a budding process. There is therefore some discrepancy in the views of Wright, and Wingrave, for the latter, "whilst admitting that a persistent exaggeration of the functions of the turbinal bodies may constitute a predisposing factor, thinks it is difficult to believe—particularly in the light of histological changes—that simple hypernutrition could be followed by any change other than hypertrophy of the vessel walls. But, given a tendency to mucous degeneration, due probably to some tropho-neurotic influences (local or general), excessive activity of the parts must play an important rôle." Wright speaks in his paper of areas of œdema, which he attributes to inflammation, but he nowhere describes any mucoid change. The subject is a difficult one, and it is not surprising that opinions on its pathology differ somewhat. Mucoid degeneration of the stroma is hard to distinguish microscopically from œdematous infiltration, and as to the changes in the walls of the sinuses, considering that the presence of a true involuntary muscular tunic is still denied by some authorities, it is apparent that the often-mentioned substitution of the muscular by fibrous tissue is not easy to demonstrate satisfactorily. That the "moriform" contour of the hypertrophies owes its origin primarily to an extension outwards of loops of distended capillaries in mucoid tissue (as implied by Wingrave), the pendulous extremities of the digitations ultimately becoming œdematous, seems to be the true fact of the case; whilst vaso motor influences and mucoid degeneration of the sinus walls occasion the distension of the cavernous spaces. In the meantime proliferation of the glandular elements, often very marked, contributes to the general hypertrophy. For further distinctions between these growths and true papillomata, and additional points in the histology of moriforms, see Pegler's paper in the transactions of the Laryngological Society of London, 1895.[3]

[1] Lancet, June 21, 1895. [2] Trans. Amer. Lar. Assoc., 1894. [3] Jrnl. of Lar. Rhin. and Otol., July, 1895.

